# Care of post-traumatic spinal cord injury patients in India: An analysis

**DOI:** 10.4103/0019-5413.36990

**Published:** 2007

**Authors:** VK Pandey, V Nigam, T D Goyal, HS Chhabra

**Affiliations:** Indian Spinal Injuries Center, Vasant Kunj, Sector C, New Delhi, India

**Keywords:** Spinal cord injury, prehospital care, treatment outcome, rehabilitation

## Abstract

**Background:**

The spinal cord injured patients if congregated early in spinal units where better facilities and dedicated expert care exist the outcome of treatment and rehabilitation, can be improved. The objective of this study is to find out the various factors responsible for a delay in the presentation of spinal injury patients to the specialized spinal trauma units and to suggest steps to improve the quality of care of the spinal trauma patients in the Indian setup.

**Materials and Methods:**

Sixty patients of traumatic spinal cord injury admitted for rehabilitation between August 2005 and May 2006 were enrolled into the study and their data was analyzed.

**Results:**

Eighty-five per cent of the spinal cord injured patients were males and the mean age was 34 years (range 13-56 years). Twenty-nine (48.33%) of the spinal injuries occurred due to fall from height. There was an average of 45 days (range 0-188 days) of delay in presentation to a specialized spinal unit and most of the time the cause for the delay was unawareness on the part of patients and/or doctors regarding specialized spinal units. In 38 (62.5%) cases the mode of transportation of the spinal cord injured patient to the first visited hospital was by their own conveyance and the attendants of the patients did not have any idea about precautions essential to prevent neurological deterioration. Seventeen (28.33%) patients were given injection solumedrol with conservative treatment, 35 (60%) patients were given only conservative treatment and seven patients were operated (11.66%) upon at initially visited hospital. Of the seven patients operated five were fixed with posterior Harrington instrumentation (71.42%) and two (28.57%) were operated by short segment posterior pedicle screw fixation. None of the patients were subjected to physiotherapy-assisted transfers or wheel chair skills or even basic postural training, proper bladder/ bowel training program and sitting balance.

**Conclusion:**

Awareness on the part of the general population, attendants of the patients, clinical and paraclinical team regarding spinal cord injury needs to be addressed. Safe mode of transportation of spinal cord injured patient and early presentation at tertiary spinal care center with comprehensive spinal trauma care team should be stressed upon.

Spinal cord injury (SCI) affects many facets of an individual's life. Often spinal cord injured patients are of the younger age group.^1^ Most of these patients are managed at centers without comprehensive spinal trauma units. The physical, personal, financial and social impact of spinal cord injury is such that most patients are lost in followup or succumb to life-threatening complications associated with spinal cord injury. However, inadequate precautions during transportation can cause further injury to the already compromised spinal cord in spinal injured patients.^2^,^3^ Early surgery and comprehensive rehabilitation markedly reduces the overall morbidity of spinal cord injured patients by enabling the patient to lead an independent life.^4^–^6^ The tertiary, regional spinal centers with the assembly of specialized trained personnel and specialized technology to provide a comprehensive rehabilitation. The larger number of patients managed in these centers permit the staff to develop greater expertise and allow more cost-effective use of resources. Furthermore, it can provide adequate number of patients for clinical trials for research in the field.^7^ Since there is no study available which discusses the problems faced by spinal cord injured patients in India, this study was conducted to assess such problems and to analyze them in order to make improvements in the present Indian setup.

## MATERIALS AND METHODS

The study was conducted between August 2005 and May 2006. Sixty patients with traumatic spinal cord injury admitted for rehabilitation were included in the study. Patients were included irrespective of the level or completeness of their spinal cord injury.

Out of 60 patients, six patients (10%) had upper cervical spine injuries (C1-C3), 10 (16.66%) patients had lower cervical spine injuries (C4-C7), 18 (30%) patients had upper dorsal spine injuries (D1-D6), 22 (36.66%) patients had lower dorsal (D7-D11) and dorsolumbar spine (D12-L1) injuries and four (6.66%) patients had lumbar and sacral spine injuries.

Out of 16 cervical spine patients 14 (87.5%) were neurologically complete (ASIA Grade A) and two (12.5%) were neurologically incomplete (ASIA Grade B). Out of 40 patients of dorsal and dorsolumbar spine injuries 34 (85%) were neurologically complete (ASIA Grade A), four (10%) patients were ASIA Grade B and two (5%) patients were ASIA Grade C. Four patients presenting with lumbar and sacral spine injuries were all ASIA Grade B (100%).

Patients were provided a questionnaire regarding their age, sex, mode of injury, delay in presentation to spinal center, cause for the delay in presentation to spinal center, mode of transportation to the first visited hospital, precautions taken during transportation to the first visited hospital and treatment they received before coming to the spinal center. Feedback data was analysed.

## RESULTS

Fifty-one (85%) of the spinal cord injured patients were males and nine (15%) were females. The mean age of males was 35 years (range 13-56 years) and the mean age of females was 31 years (range 16-38 years). The mean age of the total study group was 34 years (range 13-56 years).

Out of 60, twenty nine (48.33%) spinal cord injuries occurred due to fall from height. Out of these 29 cases, nine (31.03%) were due to fall from an unprotected terrace, six (20.68%) were due to fall from a tree, five (17.2%) due to fall in unprotected well, four (13.79%) due to fall from mountain, three (10.3%) due to fall from electric pole and two (6.8%) due to fall in drunken state from staircase. Road traffic accidents contributed for 26 (43.33%) cases. Fall of heavy object on the back accounted for four (6.6%) cases. Out of four spinal injured patients sustained following fall of heavy object on the back, three (75%) cases were due to fall of wall of the house on the back. Bullet injury accounted for one (1.6%) case [Table 1].

**Table 1:** Mechanism of injury

Mechanism of injury	Number of patients	Percentage of patients
Fall	29	48.33
Road traffic accident	26	43.33
Fall of heavy object on back	04	6.6
Bullet injury	01	1.6
Total	60	100

There was a mean 45 days (range 0-188 days) of delay in presentation. In 48 (80%) cases the patients and the attendants did not have any information regarding the existence of spinal units. In six (10%) cases financial constraint was the reason for delay in presentation, whereas in six (10%) cases, seeking treatment for associated polytrauma led to delay in presentation. Three (5%) patients sought treatment at the spinal center on the date of trauma.

A spinal injured patient was managed at a mean of 2.05 hospitals (range 0-6 hospitals) before coming to the spinal center. These hospitals ranged from local nursing home, {42 patients (70%)}, primary level health center, {35 patients (58.33%)} secondary level health center, {28 patients (46.66%)} to tertiary level health centers {18 patients (30%)}.

In 38 (63.33%) cases, the mode of transportation of the spinal cord injured patient to the first visited hospital was by their own conveyance. Out of them 18 cases (47.36%) were transported by car, 10 (26.31%) by auto rickshaw, eight (21.05%) by bullock carts and two (5.2%) by motorcycle. Hospital ambulance service accounted for the transportation of 15 (25%) spinal cord injured patients. Seven (11.66%) of the spinal injured cases were transported by police patrol van. In 49 cases (81.66%) attendants of the patient or the transporting authority did not have any knowledge about precautions essential for transportation of spinal cord injured patients to prevent neurological deterioration, as evident from the lack of precautions taken during their transport.

Seventeen (28.33%) patients were given injection solumedrol along with conservative treatment and 36 (60%) patients were given only conservative treatment at initially visited hospital. The remaining seven patients were operated, out of whom none were given solumedrol at the initial hospital (probably because of delay of more than 8h after injury). As evident from the prescription carried by the patients and from enquiring the attendants of the patient, out of the 17 patients who received injection solumedrol eight patients (47.05%) got 2g stat dose of solumedrol injection, five (29.41%) patients got 1.5g stat dose of solumedrol and two (11.76%) patients got 1g stat dose of solumedrol. Only six (35.29%) of the above patients received the drug within eight hours of trauma. Only two patients (11.76%) got loading dose on the day of trauma and the booster dose continuing the next day. Out of the 36 patients treated conservatively without solumedrol, 30 patients (83%) had managed to report to the initial hospital of contact within 8h of injury.

The patients were nursed on water mattresses, with instructions of absolute bed rest for dorsolumbosacral injuries {37 patients (61.66%)}. Sixteen patients with cervical spine injuries were managed with head halter (n = 8,50%), crutchfield tong, (n = 4,25%) and cervical collar, (n = 4,25%). Eighteen patients (30%) presenting from tertiary level health center were also instructed for regular two-hourly change of posture and chest physiotherapy.

Seven patients with lower dorsal and dorsolumbar spine injuries were operated upon with posterior Harrington instrumentation (n = 5,71.42%) and short segment posterior pedicle screw fixation (n = 2,28.57%). These included three patients who reported to the spinal centre on the day of trauma (two patients underwent pedicle screw fixation and one underwent Harrington fixation). Four other cases of Harrington fixation were done at the medical college where these patients sought treatment before coming to the index hospital for rehabilitation. None of the cervical spine injured patients were operated at the previous hospital which included several cases managed in tertiary health centers.

## DISCUSSION

Management of spinal cord injured patients in spinal units with dedicated experts and facilities for comprehensive rehabilitation improves the outcome.^8^ Comprehensive rehabilitation team of spinal trauma units consists of a doctor, a nurse, a physiotherapist, an occupational therapist, a remedial gymnast, a speech therapist, a prosthetist, an orthotist, a psychologist, a vocational evaluator and a social worker.

Males were found to be more prone for spinal cord injury in our series, which is similar to findings in other studies as they are more engaged in outdoor work and hence are more prone for spinal cord and/or other trauma.^1^,^3^,^5^,^8^,^9^–^11^ Our study also reflects the adult population being the most susceptible for spinal injuries. Adult people are the active age group of any community, which makes them more susceptible for spinal cord injuries. The mean ages of patients of SCI reported are from 30.9 years - 38.9 years from various series.^2^,^6^,^10^,^11^,^12^

Otom *et al*.,^9^ reported that in Jordan, most spinal cord injuries resulted from road traffic accidents, whereas fall from height was the third most common cause, behind bullet injuries. In Nigeria also, the most common cause for spinal cord injury was road traffic accidents, as reported by Solagberu.^10^ In our study the most common cause for spinal injuries was fall from height followed by road traffic accidents. In 19 of the 29 cases the fall could have been prevented. Most of the Indian houses in rural and urban areas lack essential safety precautions like fencing of the terrace and guarding of the staircase, thereby making fall from height a realistic possibility. Moreover, habits of sleeping on an unprotected terrace can lead to fall from the terrace while sleeping. Wells in rural India more often than not lack essential safety precautions thus putting the people working in close range at risk. Use of substandard material in the construction of rural houses (most often mud is used to construct walls of houses) endangers the lives of the people living in them. Lack of strict implementation of traffic rules in various non-metropolitan cities of India along with lack of awareness among the general population regarding adherence to traffic rules still prevails as an important cause of road traffic accident and spinal trauma.

In the United Kingdom, the average time between injury and referral is 5.5 days (range 0-94 days) and between referral and admission is 10.7 days (range 0-130 days).^5^ In Nigeria, there is delay of seven days in presentation of spinal injured patients to a spinal unit.^8^ Early presentation of spinal injured patients to a spinal unit enables early surgery, which is beneficial in terms of reducing complications, length of stay and hospital cost.^6^ Scivoletto^4^ concluded that spinal injured patients presenting early at spinal unit show better functional outcome after rehabilitation than patients presenting late. It is obvious from the results of the study that spinal cord injured patients in India present quite late at specialized spinal units and the cause for the delay in most cases was unawareness on part of patient/patient's attendants regarding existence of such spinal units. Delayed presentation to spinal units adversely affects the final functional outcome of the patient after rehabilitation.^4^

Burney *et al.*,^3^ concluded that spinal cord injured patients can be safely transported by air or ground using standard precautions. They also suggested that the distance and extent of associated injuries are the best determinants of mode of transport.^3^ The study also concluded that skeletal traction does not appear to be a prerequisite for safe, early transfer of spinal cord injured patients.^3^ Expeditious and careful transport of patients with acute cervical spine or spinal cord injuries should be carried out from the site of injury by the most appropriate mode of transportation available to the nearest capable definitive care medical facility.^2^ In a study conducted by Burney *et al.*,^3^ it was reported that more than half of spinal cord injured patients were transported by air, in Michigan. In our study we found that patients were taken to nearby hospital by their own conveyance most of the time. Moreover, attendants accompanying such patients had little knowledge regarding precautions to be taken to prevent further neurological deterioration during transportation.

Our study revealed that spinal injured patients usually shunt between a few hospitals (mean 2.05, range 0-6) before coming to specialized spinal units, thus adding damage to an already compromised spinal cord.

A study conduced by Yu *et al.*,^12^ reported that early repeated methylprednisolone sodium succinate treatment might allow greater recovery from acute spinal cord injury. Sharma *et al.*^13^ suggested that methylprednisolone sodium succinate was effective in promoting post-traumatic clinical and histological recovery and to a greater extent, when given 1h after trauma. Methylprednisolone sodium succinate is more effective than dexamethasone in reducing edema when both are given after an interval of 1h.^13^ In our study we found that only about one in four spinal cord injured patients received injection solumedrol in first visited hospital and even fewer numbers of patients got the injection in proper recommended doses.

The general population should be made aware regarding traffic safety measures, with upgradation of traffic control systems across the country. People should be made aware of the precautions that should be taken while building their houses. Banners and hoardings, pamphlets and use of various media can be very helpful in this regard. Recommendations from a study conducted by Surkin *et al.*,^11^ for prevention of spinal cord injury, like increase in safety belt usage, increasing alcohol awareness and reducing violence should also be implemented in India. Print, electronic and radio media should be used to emphasize prehospital care, precautions for transportation of spinal cord injured patients, and availability of tertiary level spinal trauma units for financially weaker sections of the society.

The government should work towards strengthening the infrastructure of primary and secondary level government hospitals for diagnosis and initial management of spinal injury patients like timely injection of solumedrol, educating the attendants of the patients regarding precautions to be taken while transporting and shifting spinal cord inured patients, creating awareness amongst villagers for spinal trauma and providing tertiary level hospitals with specialized spinal trauma units with comprehensive care for spinal injury patients.

Medical and paramedical staff across the country are still quite unaware about the socioeconomic impact of spinal cord injuries. Training programs to give an opportunity to health workers to improve their knowledge in the comprehensive management of spinal cord injured patients should be carried out on a regular basis.^14^ Hospitals managing spinal cord injured patients must have a comprehensive spinal trauma patient rehabilitation team. Complications associated with spinal cord injuries must be addressed immediately with help of specialists of other specialities like gasteroenterology, urology, plastic surgery, and general medicine. Every effort should be made to make spinal cord injured patients independent and put them back into mainstream.

Air transport of spinal injured patients has shown good results in western countries but is difficult to implement in India because of financial constrains. A suggestion from a study conducted by Nwadinigwe *et al.*,^8^ stressed upon the need to congregate spinal injured patients into regional spinal units. The study also suggested a social legislation, which will be fundamental to the social reintegration of these spinal cord injured patients. In India also, these suggestions should be given due importance so that spinal cord injured patients, after proper rehabilitation, can become part of the mainstream population.

There is a need to set up more specialized spinal trauma units across the country with good accessibility to poorer sections of society for comprehensive management of spinal cord injured patients. Early liaison of hospitals without specialized spinal units to specialized spinal centers should be encouraged, so that early presentation of acute spinal cord injured patient to a specialized spinal unit leading to early surgery and comprehensive rehabilitation can be carried out.^4^,^5^ Kishan *et al.*,^6^ emphasized that early surgical treatment is beneficial in terms of reducing complications, length of stay and hospital costs.^6^ Fehling and Perrin^15^ suggested that urgent decompression in acute cervical spinal cord injury remains a reasonable practice option and can be performed safely. Early decompression and stabilization of injured spinal cord is an area that is still overlooked in the Indian setup.

This is quite evident from our study in which only 11.6% of the patients were decompressed and stabilized. None of the above patients included cervical spine injury, a group for which early mobilization is all the more important to prevent complications.

Job reservations should be encouraged for spinal cord injured patients. The home visit program conducted at Ahmedabad by Prabhaka and Thakkar^16^ for spinal cord injured patients decreased the number of re-admissions by improving the status of rehabilitation, which raised the quality of care for patients with spinal cord injury. Such programs can be carried on a broader basis like national programs, so that maximum numbers of spinal cord injured patients are benefitted. The government should take measures to improve the management of spinal injured patients including health education on passenger and load carriage, promoting use of manual or motorized wheel barrow as against bearing heavy load on the head, principles of moving spinal injured patients taught to general population, establishment of government-aided spinal centers and training of specialized personnel.^10^

Our study has the limitation that it was conducted at a tertiary level spinal center in a metropolitan city; the situation may be more grave in other parts of India.

## CONCLUSION

There is an urgent need to take steps to prevent spinal cord injury, strengthen the prehospital care transportation network and treatment in specialized spinal trauma units to avoid loss of young active manpower. There is need to increase tertiary spinal trauma units where there is multidisciplinary approach for comprehensive rehabilitation service of spinal cord injured patients.
